# ErmineJ: Tool for functional analysis of gene expression data sets

**DOI:** 10.1186/1471-2105-6-269

**Published:** 2005-11-09

**Authors:** Homin K Lee, William Braynen, Kiran Keshav, Paul Pavlidis

**Affiliations:** 1 Columbia Genome Center, Columbia University, New York NY 10032, USA; 2 Department of Computer Science, Columbia University, New York NY 10025, USA; 3 Department of Philosophy, University of Arizona, Tucson, AZ 85721, USA

## Abstract

**Background:**

It is common for the results of a microarray study to be analyzed in the context of biologically-motivated groups of genes such as pathways or Gene Ontology categories. The most common method for such analysis uses the hypergeometric distribution (or a related technique) to look for "over-representation" of groups among genes selected as being differentially expressed or otherwise of interest based on a gene-by-gene analysis. However, this method suffers from some limitations, and biologist-friendly tools that implement alternatives have not been reported.

**Results:**

We introduce ErmineJ, a multiplatform user-friendly stand-alone software tool for the analysis of functionally-relevant sets of genes in the context of microarray gene expression data. ErmineJ implements multiple algorithms for gene set analysis, including over-representation and resampling-based methods that focus on gene scores or correlation of gene expression profiles. In addition to a graphical user interface, ErmineJ has a command line interface and an application programming interface that can be used to automate analyses. The graphical user interface includes tools for creating and modifying gene sets, visualizing the Gene Ontology as a table or tree, and visualizing gene expression data. ErmineJ comes with a complete user manual, and is open-source software licensed under the Gnu Public License.

**Conclusion:**

The availability of multiple analysis algorithms, together with a rich feature set and simple graphical interface, should make ErmineJ a useful addition to the biologist's informatics toolbox. ErmineJ is available from .

## Background

A difficulty experienced by many (if not all) users of gene expression microarrays is making sense of the complex results. After analyzing each gene in a data set, an experimenter is often left to the task of summarizing the results with little assistance. It is common for experimenters to ask questions at the level of molecular pathways or other functionally relevant groupings of genes. While "ad hoc" manual annotation of data sets is a common approach, there are numerous advantages to using a computational and statistical approach to analyze groups of genes.

The most common means of performing this analysis is to ask whether certain Gene Ontology (GO) [1] terms are "over-represented" in a set of genes selected by fold-change or statistically-motivated approaches such as a t-test. This is easily implemented by using the properties of the hypergeometric distribution (often referred to as Fisher's exact test for two categories) or its binomial approximation. In our work, these methods are more generically referred to as "over-representation analysis" or ORA. In addition, as the GO is just one way of organizing genes, we refer to the general goal of these methods as "gene set analysis", where a gene set is any grouping of genes not derived from the data itself, typically based on biologically-motivated criteria.

The need to perform ORA has led to the emergence of a variety of tools. A list of many such tools is available from the Gene Ontology Consortium [2], and a large number of them were recently reviewed [3]. However, to our knowledge these tools all implement ORA methods; other methods or algorithms are not available, with the exception of the Perl script Catmap [4]. Thus these tools primarily differentiate themselves through user interface features, ease of use, supported data types, and speed [3]. Most tools surveyed by [3] were reported to have one or more significant limitations, including slow performance, an inability to analyze gene annotations other than those directly annotated (that is, other levels of the GO hierarchy are not considered), requiring web access to use, are difficult to install (limiting their usefulness to biologists), or lack the ability to visualize the GO hierarchy [3].

In this paper we describe ermineJ, a stand-alone tool that implements methods described by [5] and [4] in addition to ORA, has a rich feature set, and does not have the limitations cited above. One of the offered analysis methods in particular is complementary to ORA analysis, which we now call Gene Set Resampling or GSR (the "experiment" score in Pavlidis et al. (2002)). In GSR, the gene-by-gene scores (e.g., t-test p-values) are not thresholded. Instead, for each gene set an aggregate score is computed, such as the geometric mean of the p-values for genes in the category, and the significance of that score determined by random sampling of the data. We have recently presented some evidence that GSR can provide better results than ORA in some situations [6].

ErmineJ also has methods for analysis of genes based on rankings (the receiver operator characteristic, or ROC) [4]. ROC can be thought of as a version of ORA where all possible thresholds are considered simultaneously. Like GSR, the ROC method utilizes non-thresholded gene scores, but considers only their ranking, which might be considered more robust than using the raw gene scores. Finally, ErmineJ offers an analysis based on the correlation of gene expression profiles, gene group correlation analysis (GCA) [5]. GCA can be used as an alternative to the use of ORA for the determination of whether genes in particular functional categories are "clustering together".

The first version of ermineJ was made available in 2003. Recently we have completely revamped the user interface and updated the feature set, releasing ermineJ 2.0 in October 2004 and 2.1 in June 2005.

## Implementation

ErmineJ is implemented entirely in the Java programming language [7]. It uses the Java Swing libraries to create a graphical user interface that can run on many different platforms. Architecturally, an effort has been made to separate analytical and algorithmic concerns from user presentation concerns. Besides being a design best practice, the architecture was also driven by the need to support command-line interfaces as well as application programming interfaces to the methods. The structure of ermineJ also lends itself to fairly easy extensibility, so new algorithms can be added to the software as requirements change. The analysis algorithms in ermineJ were previously described [4,5].

In addition to using the Java SDK, ermineJ depends on a number of free third-party libraries, most notably the Colt library [8]. Colt is a high-performance numerical computing library that includes implementations of many linear algebra and statistical methods, as well as useful data structures which we rely on heavily in our software. Other libraries ermineJ uses include various Jakarta Commons libraries [9], and the Xerces XML parsing engine [10], which we use to parse the Gene Ontology XML description. Many of the low-level numerical and utility routines (e.g., for file parsing and string manipulation) are tested in an extensive unit test suite.

## Results and discussion

### Inputs

All interfaces to ermineJ use the same basic inputs. The first is a description of the Gene Ontology in XML format, obtained from the GO consortium web site [11]. The second is a description of the microarray platform (the "array annotation file", which contains tab-delimited text), which associates probe identifiers with Gene Ontology terms and additionally associates each probe with a gene (used in the statistical analysis to account for repeated genes, as described below) and descriptions that are useful for viewing in the context of the results. The third required input is the user's own data. For ORA, GSR and ROC applications, this takes the form of a list of gene scores, one for every probe set on the array design. Alternatively (for expression profile correlation analysis), the input can be the expression profile matrix, as might be used as an input to a clustering tool. The gene scores can be p-values or another score such as fold-change. ErmineJ is purposefully largely agnostic about the meaning of the gene scores, and focused on the distributional properties of the scores.

We maintain on the order of 30 different mouse, human and rat array annotation files for different platforms, as well as generic files for RefSeq [12] genes that can be used to construct annotation files for other platforms (available from our web site [13]). The native annotation file format is very simple and new files can easily be constructed with a modicum of bioinformatics skill. ErmineJ can also read Affymetrix "CSV" (comma-separated-value) annotation files available from the manufacturer's web site. We gladly entertain requests to add support for other arrays. When an annotation file is read in, the software automatically associates each probe with all parent terms of each directly annotated terms. For example, all genes annotated with the term "regulation of cell size" are also associated with the higher-level terms "cellular morphogenesis" and "morphogenesis". This feature is only supported by some of the tools reviewed by [3].

There are a number of parameters to set and decisions the user must make in order to run the software. The choice of analysis method is the most obvious, and each method has a few other settings that the user can choose to change. For example, for ORA analysis a threshold score must be defined. This is in contrast to most ORA software packages which take as input a list of "genes of interest"; instead, ermineJ takes as input all the gene scores for the experiment. This lets ermineJ avoid the problem of selecting the correct "null" gene set [3]: it is defined strictly by the genes analyzed in the experiment but not meeting the user-defined score threshold.

For GSR, the method used to compute the score for a gene set is a key parameter. The two options currently supported are the mean and the median. During the analysis, GSR uses the selected method to compute a summary of the gene scores for each resampled or real gene set, and this aggregate score is used to represent the gene set. Choosing the median will tend to yield slightly more conservative results, as individual genes with very high scores are not given as much weight as in the mean computation.

Some settings are used for multiple methods. For example, when a gene is represented more than once in the data set, a decision has to be made as to how to treat these "replicates" (which might not be replicates *per se *but represent different transcripts). The options supported are to use the "best" score among the replicates to represent them as a group; to use the mean; or to treat them as separate entities. Use of the "best" option is somewhat anti-conservative, but is reasonable when most "replicates" are in fact assaying different biological entities. In contrast, treating replicates completely separately is not generally advised as it can lead to spurious positive findings in cases of true replicates, as the gene set gets "adulterated" with multiple copies of the same high-scoring gene. For this reason the last option is not available from the GUI, though it can be accessed from the other interfaces. Another important setting is the range of gene set sizes to analyze. Gene sets that are very small are unlikely to be very informative, because the goal of the analysis is to study genes in groups, while large gene sets may be too non-specific to provide useful information. In addition, analyzing too many gene sets reduces the power of the analysis due to multiple testing costs. In practice we often use a range of 5–100 or 5–200.

In addition to the pre-defined gene sets as defined by the Gene Ontology, users are free to input their own gene sets. These are defined in simple text files that are placed in a directory that ermineJ checks at startup. These text files can be created "off-line" or within the ermineJ GUI. In addition, users can modify gene sets from within ermineJ. This functionality can be used to correct errors or omissions in the Gene Ontology annotations, though care must be exercised to avoid introducing biases into the results.

### Types of analysis

#### Gene-score based methods

The ORA, GSR and ROC methods are closely related in that they are based on the gene-by-gene scores, with the goal of finding gene sets that are some sense "enriched" in high-scoring genes (which typically might be "differentially expressed genes"). ORA is sometimes used to analyze genes which are selected by clustering, rather than a continuous score. In this situation, GSR and ROC are not appropriate. However, the correlation method is specifically designed to address this situation. GSR and ROC have the benefit of not requiring a threshold to divide genes into "selected" and "non-selected" genes. The choice of the threshold for ORA can have a substantial effect on the results obtained, because the "selected genes" change [4].

#### Correlation analysis

Gene group correlation analysis (GCA) is based on the similarity of the expression profiles of genes in a gene set: loosely speaking, how well they "cluster together". Thus we propose that GCA can be used as an alternative to using ORA to analyze clusters. There are some differences to be noted between the typical application of ORA to clusters and the ermineJ correlation analysis. GCA is group-centric, not cluster-centric. Thus we ask whether the correlation among the members is higher than expected by chance, not whether a given set of correlated genes is enriched for the genes in the group; GCA does not involve clustering. This is not a trivial distinction, because while the highest scores will be obtained for gene groups that have uniform and high correlations among all the members, groups that have two or more "sub-clusters" can also obtain high scores. In the current implementation of GCA, the absolute value of the correlation is always used, which allows. In future versions we may expose this as a user-settable option, as well as implementing other possible correlation metrics other than the current Pearson correlation.

In all methods, for each gene set analyzed, ermineJ computes a score and, based on that score and the gene sets size, a p-value representing the "significance" of that gene set with respect to the null hypothesis. The definition of the raw score and the null hypothesis depends on the method being used. Note that the raw scores are of limited use because it cannot be evaluated in the absence of information about the gene set size. However, they can provide the user with a helpful indication the strength of the result, not just its statistical significance.

For ORA, the null hypothesis is that the genes in the gene set are distributed randomly between the "selected" genes and the "non-selected" genes. The raw score reported by ErmineJ is the number of genes in the set which pass the threshold for gene selection. For GSR, the null hypothesis is that the mean (or median) gene score (which forms the gene set score; for p-values negative-log-transformed values are used) is drawn from the global (data-wide) distribution of possible gene set mean (or median) gene scores, as determined by resampling [5]. For ROC analysis, the null hypothesis is that the genes in the gene set are distributed randomly in the ranking; p-values are computed using the fact that the ROC is equivalent to the Wilcoxon rank-sum test [4]. The raw gene set score is simply the area under the receiver operator characteristic curve [14], which ranges from values of 0.5 (random ranking) to 1.0 (all genes in the gene set at the top of the ranking). Finally, for correlation analysis, the null hypothesis is that the mean pairwise correlation of profiles in the gene set is drawn from the global distribution of gene set correlation scores, as determined by resampling [5]. The raw score is the mean absolute value of the pair-wise correlation of the genes in the set (comparisons of a probe to itself, or to other probes for the same gene, are always ignored).

ErmineJ includes implementations of three multiple test correction methods (though currently only one of these, Benjamini-Hochberg false discovery rate (FDR) [15], is made available through the GUI). The additional options, available from the command line, are Bonferroni correction and a resampling-based family-wise error rate correction [16]. The FDR is used in the GUI as a rapid and reasonable guide to which gene sets are likely to be of highest interest.

### The ermineJ GUI

Most users of ermineJ will access it through its graphical interface. The GUI of ermineJ was designed to be simple to use and provides "wizards" to guide users through common tasks such as running an analysis. Many settings made by the user during operation of the software are remembered between sessions, facilitating repeated analysis of the same data files and maintaining the user's preferred window sizes, for example. A complete manual is provided and is accessible via an on-line help function, as web pages on our web site, or in portable document format (PDF).

**Figure 1:**
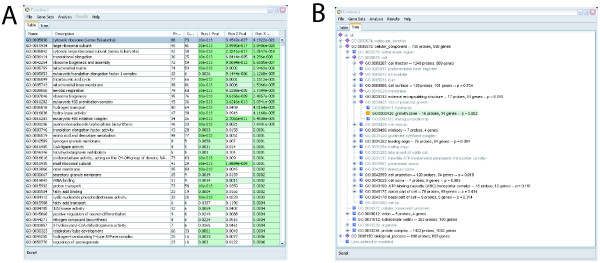
**A: **The main panel of ErmineJ after several analyses have been performed. Gene sets selected at low FDR levels are indicated in color. **B: **The tree-view panel of ErmineJ, illustrating the ability to browse gene sets in the GO hierarchy. The icons at each node have specific meanings. For example, the yellow "bull's-eye" icon indicates a gene sets selected at an FDR of 0.05 or less. Purple diamonds indicate nodes that have "significant" sub-nodes.

**Figure 2:**
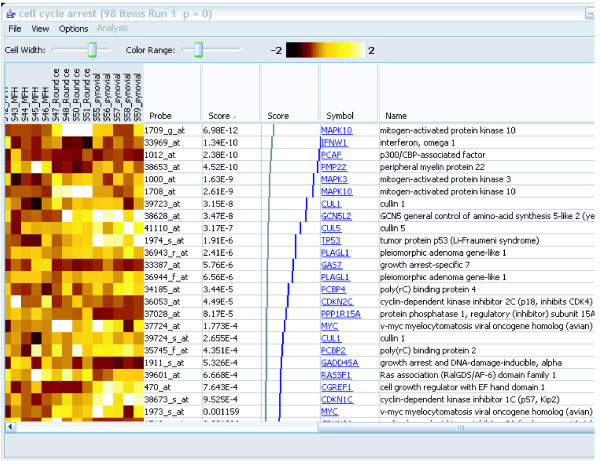
A gene set details view. The controls at the top allow adjustment of the size and contrast of the heat map. The gene scores (in this case p-values) are shown in the second text column. The grey and blue graph, shown only for experiments using p-values, shows the expected (grey) and actual (blue) distribution of p-values in the gene set. This display is provided as an additional aid to evaluation of the results. The last two columns provide information about each gene. The targets of the hyperlinks are configurable by the user.

**Figure 3:**
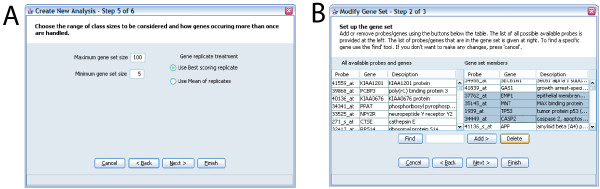
Examples of screens from ErmineJ Wizards. **A: **Analysis wizard. This illustrates options to set the range of gene set sizes to analyze, and the method of treating "replicates" of genes. See text for details of the latter. **B: **Gene set modification wizard. In this screen the user is selecting genes to delete from a gene set. The list of all probe available on the platform is available in the left panel. A "find" function simplifies the location of genes and probes.

Some aspects of the ermineJ graphical user interface is illustrated in Figures 1, 2, 3. The main panel of the software can be viewed either as a table of gene sets (Figure 1A) or in a hierarchical (tree) view (Figure 1B). These views are linked so changes in one are reflected in the other. To facilitate navigation of these displayed, gene sets can be searched by the name of the gene set or by the names of genes they contain. User-defined gene sets are displayed in contrasting colors. Not shown in the figures is the initial startup screen in which the user chooses the gene annotation file to use for the session.

Double-clicking on a gene set in the main panel opens a new window that displays the genes in the gene set, along with the expression profiles in a "heat-map" view (if the user has provided the profile data; Figure 2). The appearance of the heat map is configurable through menus and toolbar controls. The data displayed in the table, as well as the image of the matrix, can be saved to disk using additional menu options. The hyperlinks to external web sites can be configured by the user to point to a web site of their choosing, again through a menu option. All of these capabilities are available even if the user has not performed any analysis, so ErmineJ can be used as a "gene set browser" as well as for analysis.

An important feature of the GUI is the capability to rapidly define and edit gene sets, which is accomplished in a "wizard" that takes the user through the process set-by-step. Alternatively, the user can simply populate the gene set directory with files they have obtained from other sources, for example created in bulk with a Python script or obtained from another user. As far as we know, no tool surveyed by [3] affords the user the ability to define or modify the categories. ErmineJ also allows the user to choose which of the GO aspects (Biological Process, etc.) to use in the analysis.

The GUI version of ermineJ can be installed on the user's computer or run via Java WebStart. The latter option simply involves clicking on a link in the user's web browser, and ensures that the users have the most up-to-date version of the software. The drawback of using WebStart is that the user must be connected to the internet to use the software. With a local installation, no internet connection is needed.

### Running an analysis

Running an analysis using the ErmineJ GUI involves using a "wizard" to set the parameters (Figure 3). The user is asked to choose an analysis method, select the data file to analyze, choose any user-defined gene sets to include in the analysis, and set the various parameters required for the particular analysis. All settings are documented via "tool tips" and in the manual.

Once an analysis is initiated, the user is informed of its progress via a status bar. An analysis can be cancelled any time. On completion, the results are added to the tabular and tree views (Figure 1). Multiple results can be displayed simultaneously in the tabular view, allowing easy comparison of different runs. The tree view can display only a single analysis result set at a time, but offers a pull-down menu to selected among the results sets to display. In the tree and tabular views, high-scoring (i.e., significant) gene sets are highlighted in color. The tree view uses a simple system of icons for each node to indicate whether a significant node is contained within a given higher level node. Finally, the results of an analysis can be saved to a tab-delimited file for use in other software or to be reloaded by ermineJ at a later time.

### Other interfaces

In addition to the GUI, ermineJ offers a command line interface (CLI) and a simple application programming interface (API). The CLI exposes some features of ermineJ that are not available in the GUI, such as different methods for multiple test correction. The CLI is suitable for scripting runs of ermineJ. For example, a simple Perl script can be used to automate runs of ermineJ with different settings or on different data sets. In contrast, the API was introduced to allow programmers to include the analyses available in ermineJ in their own software. The API currently provides more limited access to the functionality of the software than the command line version, but will be expanded in future versions.

### Performance

We tested the performance of ermineJ using the HG-U133_Plus_2 Affymetrix array design. This is a particularly large array design with over 54,000 probe sets, and represents a something of a worst-case scenario with respect to performance. With our current annotation set, 4844 different GO categories (gene sets) are available for analysis in this array design. We limited our analysis to gene sets with between 5 and 100 genes, leaving about 2700 gene sets. The times reported below are for analyzing the complete set of over 54,000 probe sets with respect to these 2700 gene sets on a on a 1.7 GHz Pentium laptop.

With this array, ermineJ has an initial startup phase that lasts 15–20 seconds, most of which is consumed by time it takes for the gene annotation file to be read in and processed for analysis. The time for analysis once startup is completed depends on the method used. For ORA, a complete analysis is completed in 8 seconds (average of 3 runs; times are wall clock seconds timed from within the software). While it is difficult to directly compare our benchmarks with previously published benchmarks because the number of gene sets analyzed and the size of the "null" gene set was not reported, and the times reported might in some cases include initial startup times [3], the fastest reported methods on the largest data sets tested completed ORA analyses in under 10 seconds. This indicates that ErmineJ is at least competitive with and possibly faster than the fastest previously reported tools.

GSR analysis took about 370 seconds if a full resampling is performed (100,000 resampling trials per gene set size in our tests). However, ermineJ implements an approximation, where limited resampling is used to estimate the parameters of a normal distribution. This normal is used to compute the p-values for each gene set. It also takes advantage that, especially for larger class sizes, the shape of the resampled distribution is very similar for similar class sizes, so not all of them need to be computed. In this mode the analysis takes approximately 80 seconds. ROC analysis, which does not involve resampling, took about 100 seconds. Correlation analysis is the most computationally intensive resampling method; even with the approximations enabled it currently takes about 400 seconds to run on the test data set (which contained 12 microarrays). This is because computing correlations is computationally intensive, compared to the methods which use pre-computed gene scores such as p-values.

ErmineJ is fairly memory-intensive, because it holds in memory a complex data structure describing the annotations, as well as the microarray data and information about the results for thousands of gene sets and tens of thousands of genes. For the large HG-U133_Plus_2 design, after startup ermineJ occupies approximately 85 Mb of RAM (determined using a Java heap profiler under Windows). After running the correlation analysis, this grew to 105 Mb, reflecting the loading of the complete expression profile set and the results. Therefore we recommend running ermineJ on machines that have at least 256 Mb of RAM.

### Future plans

At this writing, the current version of ermineJ is 2.1.6. New features planned for the software include expanding the API and allowing more flexible creation of user-defined gene sets, including allowing support of alternative nomenclatures such as the Plant Ontology [17]. We also plan to provide annotation files for more platforms and organisms.

We have been interested in the possibility of including other resampling-based methods such as GSEA [18] or the similar resampling method implemented in Catmap [4] in ermineJ. The primary reason to consider these methods is that they examine the distribution of gene scores by resampling over the samples, which is more correct than merely resampling over the genes. This is because the null hypotheses in the gene score analysis are some variation on a random distribution of genes within the ranking of genes. This assumption can be badly violated for gene sets containing highly correlated genes (such as the ribosomal protein genes); such genes will tend to have correlated rankings, and in some situations (particularly when the gene p-value distribution is close to uniform), spurious false positives can occur [4]. The ORA, GSR and ROC methods are all susceptible to this problem, though we stress that this is only an serious issue for gene sets that show high correlations not related to the experimental design.

It would be challenging to provide a general-purpose implementation of GSEA or Catmap that is easily accessible to biologists with limited computational skills. These methods require either that users can provide the gene scores for hundreds (if not thousands) of resampled data sets [4], a task that is difficult to accomplish for the targeted user base of ermineJ, or computation of gene scores by the software. Because each experimental design might have a different mechanism for computing gene scores (fold-change, t-test, ANVOA, Cox regression, etc), it would be difficult to provide a fully flexible tool without including a full-fledged statistical analysis package as well. A feasible solution we are considering is to cover the most frequently-encountered situations (e.g., t-test and one-way ANOVA).

## Conclusion

ErmineJ is a fast, full-featured, user-friendly, multi-platform open source application for analysis of gene sets. It implements multiple algorithms for performing the analysis, and permits easy modification and creation of new gene sets. These features afford users considerable flexibility in testing different methods and parameters. Perhaps the greatest current limitation to its usability at this date is the availability of gene annotation files for non-Affymetrix array designs we have not encountered frequently. Users who wish to develop annotation files for their platform should contact us for assistance.

## Availability and requirements

• **Project name: **ErmineJ

• **Project home page: **

• **Operating system(s): **Platform independent

• **Programming language: **Java

• **Other requirements: **Java 1.4 or higher; 256 Mb RAM recommended.

• **License: **GNU GPL and LPGL for helper library.

• **Any restrictions to use by non-academics: **None

## List of Abbreviations

ORA: Over-representation analysis

GSR: Gene score resampling

ROC: Receiver operator characteristic

GCA: Gene group correlation analysis

GSEA: Gene Set Enrichment Analysis

FDR: False discovery rate

GO: Gene Ontology

GUI: Graphical User Interface

API: Application Programming Interface

CLI: Command Line Interface

## Authors' contributions

PP was the project lead and chief architect of the software, and contributed to the source code. HKL, WB and KK all contributed to the source code.
